# Two Years Beyond COVID-19: Unveiling the Persistence of Neuropsychiatric Symptoms and Risk Factors in a Cross-Sectional Study

**DOI:** 10.1192/bjo.2024.189

**Published:** 2024-08-01

**Authors:** Poulami Laha, Guru S Gowda, V Senthil Kumar Reddi, Harish T, Sydney Moirangthem

**Affiliations:** 1 National Institute of Mental Health and Neurosciences, Bengaluru, India

## Abstract

**Aims:**

The neuropsychiatric morbidities associated with post-COVID status are important public health issues. The range and severity of morbidity varies with the type of clinical setting and time of assessment. There are limited studies on the long-term persistence of the post-COVID neuropsychiatric symptoms (PCNS). Hence, this study aims to determine the proportion of persistent PCNS after approximately 2 years of COVID and to find any risk factors for persistent PCNS.

**Methods:**

This study was a cross-sectional study of randomly selected 2,281 individuals aged 18–60 years, currently living in the community, who were RT-PCR positive for COVID-19 from the National Institute of Mental Health and Neurosciences (NIMHANS) laboratory (at least 4 weeks before intake) from a period of 1 June 2020 to 31 March 2022. Among them, 927 individuals who met the study criteria were screened for PCNS through telephone interviews using a validated PCNS screening tool comprising sociodemographic details, life events inventory and 20 questions to assess for PCNS. 196 individuals who came positive for PCNS were further evaluated by in-person or web-based interviews with Structured Clinical Interviews for DSM–5-Research Version and World Health Organization-Post-COVID Case Report Form for persistent PCNS. Descriptive statistics, Chi^2^ test, Mann–Whitney U Test, and Binary logistic regression analysis were used for data analysis. The Institutional Ethics Committee approved this study.

**Results:**

The median age of study participants was 34 years, and 51.3% were female. 68 out of 196 participants (34.7%) had persistent PCNS approximately 2 years (23.84 months) after COVID-19 infection. Chronic fatigue (10.2%), depression (6.1%), cognitive symptoms (4%), hyposmia (3.6%), hypogeusia (3.6%), anxiety (2.5%), panic disorder (2.5%) and insomnia (2%) are the main persistent symptoms. The median age of the participants with persisted PCNS (40 years) is higher compared with the median age of the participants without persisted PCNS (34 years) [Mann–Whitney U = 5,225.0, P = 0.021]. Even though significant associations were found between the development of PCNS after 4 weeks of COVID and female gender, symptomatic COVID-19, severity of COVID-19 (oxygen supplementation), hospital admission, total number of times of COVID-19, and presence of life events, this association were not found with persistence of PCNS at 2 years.

**Conclusion:**

This study revealed that one-third of the individuals with PCNS had persistent symptoms after 2 years. Chronic fatigue is the most common persistent PCNS. Middle-aged and above age groups were found to be a risk factor for persistent PCNS.

